# A novel approach for resolving differences in single-cell gene expression patterns from zygote to blastocyst

**DOI:** 10.1093/bioinformatics/bts385

**Published:** 2012-09-03

**Authors:** Florian Buettner, Fabian J. Theis

**Affiliations:** ^1^ Institute of Bioinformatics and Systems Biology, Helmholtz-Zentrum München, 85764 Neuherberg, Germany; ^2^ Department of Mathematics, TU München, 85748 Garching, Germany

## Abstract

**Motivation:** Single-cell experiments of cells from the early mouse embryo yield gene expression data for different developmental stages from zygote to blastocyst. To better understand cell fate decisions during differentiation, it is desirable to analyse the high-dimensional gene expression data and assess differences in gene expression patterns between different developmental stages as well as within developmental stages. Conventional methods include univariate analyses of distributions of genes at different stages or multivariate linear methods such as principal component analysis (PCA). However, these approaches often fail to resolve important differences as each lineage has a unique gene expression pattern which changes gradually over time yielding different gene expressions both between different developmental stages as well as heterogeneous distributions at a specific stage. Furthermore, to date, no approach taking the temporal structure of the data into account has been presented.

**Results:** We present a novel framework based on Gaussian process latent variable models (GPLVMs) to analyse single-cell qPCR expression data of 48 genes from mouse zygote to blastocyst as presented by (Guo *et al.*, 2010). We extend GPLVMs by introducing gene relevance maps and gradient plots to provide interpretability as in the linear case. Furthermore, we take the temporal group structure of the data into account and introduce a new factor in the GPLVM likelihood which ensures that small distances are preserved for cells from the same developmental stage. Using our novel framework, it is possible to resolve differences in gene expressions for all developmental stages. Furthermore, a new subpopulation of cells within the 16-cell stage is identified which is significantly more trophectoderm-like than the rest of the population. The trophectoderm-like subpopulation was characterized by considerable differences in the expression of Id2, Gata4 and, to a smaller extent, Klf4 and Hand1. The relevance of Id2 as early markers for TE cells is consistent with previously published results.

**Availability:** The mappings were implemented based on Prof. Neil Lawrence's FGPLVM toolbox^1^; extensions for relevance analysis and including the structure of the data can be obtained from one of the authors' homepage.^2^

**Contact:** f.buettner@helmholtz-muenchen.de

## 1 INTRODUCTION

**Fig. 1.**
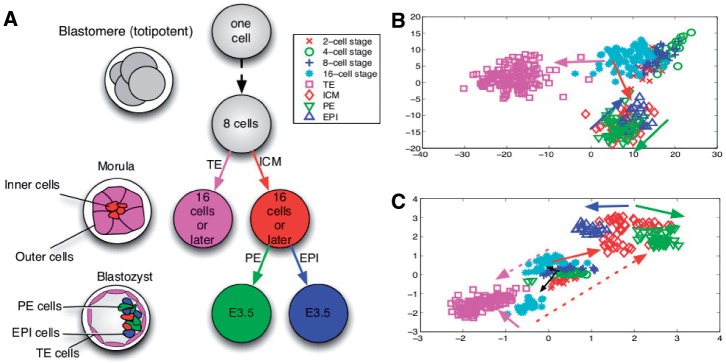
The totipotent blastomere differentiates first into inner and outer cells. Next, after approximately 3.5 days, the ICM differentiates into PE cells and EPI cells (**A**). The data-driven illustration is shown on the right hand side. For PCA (panel B), differentiation into ICM and TE can be seen, followed by differentiation from ICM into PE and EPI. ICM and PE/EPI as well as early cell stages could not be resolved. For our novel approach (bottom right), all developmental stages could be resolved and a new TE-like sub-population at the 16-cell stage was discovered. The dashed arrows reflect that the lower subpopulation at the 16-cell stage is significanlty more TE-like than the other

During embryonic mouse development, the initially totipotent 1-cell zygotes become restricted in their potential and through a sequential differentiation process, different lineages are generated. Differentiation of embryonic stem cells of the mouse is thought to start in the 8-cell stage (Johnson and McConnell, 2004). First, differentiation between inner cell mass (ICM) and trophectoderm (TE) can be observed. The TE gives rise to extra-embryonic structures such as the placenta while the ICM subsequently differentiates into primitive endoderm (PE) and the epiblast (EPI). Although the PE will also give rise to extra-cellular structures providing nutrient supplies for the embryo, the pluripotent EPI gives rise to the foetus (Fig. 1). For a better understanding of the mechanisms and timing of cell fate decisions, it is desirable to assess gene expression patterns at different stages of the developing cells. Conventional techniques measure these gene expressions from pools of cells; however, as fate decisions are made by individual cells, this may mask the dynamics of single cells (Guo *et al.*, 2010). Recent technical advances allow for measuring the expression of multiple genes in single cells by means of a quantitative polymerase chain reaction (PCR) method (Taniguchi *et al.*, 2009). Such methods are a promising tool which will provide a new wealth of data in the future. To gain insights in underlying fate decisions, it is important to develop computational techniques allowing for a comprehensive analysis of this new type of data. In this article, we present our new approach at the example of the cellular development of the mouse zygote to blastocyst; however, our methodology is not limited to this particular example and can be applied to a range of other datasets and cell types.

The most comprehensive analysis to date linking cell fate decision during the development of the zygote to the blastocyst to gene expression data has been performed by (Guo *et al.*, 2010). The authors analysed the expressions of 48 genes in single cells. Therefore, the expression levels of these 48 genes were measured at different stages of the cellular development (from 1-cell stage of the mouse embryo to 64-cell stage) on a single-cell level. (Guo *et al.*, 2010) have analysed these data by performing a PCA of expressions of 48 genes for cells at the 64-cell stage. Although it was possible to use results from the PCA to resolve transcriptional differences between TE, PE and EPI cells, no differences in gene expression patterns for cells until the 16-cell stage could be resolved using PCA. Although this analysis provided valuable insights into cell fate decisions after the 16-cell stage, the authors reported that no differences in gene expression patterns could be found for earlier stages in the cell development. In a subsequent analysis, the authors identified Id2 and Sox2 as the earliest markers for differentiation of outer and inner cells followed by inverse correlations of Fgfr2/Fgf4 in the inner cell mass. However, Id2 and Sox2 could only be identified by a univariate analysis of the distribution of gene expressions within the 16-cell stage and the 32-cell stage. Although PCAproved to be a useful technique to identify some markers for TE, PE and EPI stage, a different approach for dimensionality reduction which embeds the high-dimensional data nonlinearly in a two-dimensional or three-dimensional space, may yield deeper insights. Thus, differentiation is thought to start as early as at the 8-cell stage (Johnson and McConnell, 2004); if this is reflected in early changes in gene expression patterns, it may be possible to identify these changes by embedding the data in a low-dimensional space. Taking into account potentially complex interrelations between different genes may result in an embedding better reflecting the true structure of the data and allowing for reliable identification of new subpopulations and markers, also in these early cell stages. However, identifying a suitable alternative to PCA is challenging for several reasons.

First, there is usually a trade-off between interpretability and complexity of an embedding: linear techniques such as PCA are usually well interpretable as those factors which are most important at specific areas of the mapping can be identified by analyzing the loadings of the PCA. In contrast, the results of many more complex, nonlinear embeddings (Sammon, 1969; Shieh *et al.*, 2011; van der Maaten and Hinton, 2008) are hard to interpret as no explicit mapping between the low-dimensional latent space and the high-dimensional data space (or vice-versa) is related to the embedding.

Second, it is not clear how the group structure of the data, i.e. the fact that different instances of the data come from one of seven different developmental stages, can be considered when performing the embedding. At one extreme, it is possible to perform one embedding of all the data pooled together without considering its structure; at the other extreme, it is possible to perform a separate embedding for each developmental stage. Both approaches have severe drawbacks, as in the first approach potentially useful information is discarded while in the latter approach similarities across cell stages are not considered.

In the following, we present a novel approach for a nonlinear, interpretable embedding of gene expression data from different developmental stages of the mouse embryo which takes the group structure of the data into account. The benefits of our approach are illustrated at the example of gene-expression data from the mouse zygote to the blastocyst, as presented in (Guo *et al.*, 2010). We show how it can allow for a comprehensive analysis of single-cell data from different groups of cells, potentially yielding better insights into cell fate decisions than previously proposed approaches.

## 2 METHODS

To capture first transcriptional differences indicating a commitment to specific cell fates, it is important to analyse gene expression patterns at different cell stages (time points in the differentiation process). Therefore, Guo *et al.* (2010) analysed mRNA levels of 48 genes in parallel. The authors performed a linear PCA of the gene expression data at the 64-cell stage for dimension reduction purposes. At this cell stage, TE, EP and EPI cells can be clearly differentiated based on the expression of known markers and can also be identified as clusters in the PCA. Next, the gene expression data for earlier cell stages were projected onto the first 2 PCs (of the 64-cell stage PCA) to assess transcriptional changes at earlier stages. No differences between the projected gene expression patterns can be seen for cell stages 2–8, and the authors report that no distinguishing characteristics among cells at the 2-, 4-and 8-cell stage could be found.

However, these conclusions were based on a linear PC analysis. To test whether nonlinear effects play a role and could allow the identification of distinguishing characteristics of gene expression patterns at earlier cell stages, a nonlinear embedding of the high-dimensional gene expression data in a low-dimensional latent space was performed. To yield an interpretable embedding, it is desirable to define an explicit mapping, either from data space into latent space (as for PCA) or from latent space into data space. Therefore, a nonlinear probabilistic generalization of PCA (Gaussian process latent variable model (GPLVM)) (Lawrence, 2004) was performed. Although a variety of other nonlinear methods for dimensionality reduction have been proposed in recent years (Shieh *et al.*, 2011; van der Maaten and Hinton, 2008), the resulting embeddings for these methods are difficult to interpret as no explicit mapping is defined.

### 2.1 Guassion process latent variable model

Let the gene expressions in the data space be denoted by *Y* = [*y*_1_,...,*y_N_*]*^T^*, *Y_i_*∈*R^D^* and latent variables in the low-dimensional latent space be denoted by *X* = [*x*_1_,...,*x_N_*]*^T^*, *X_i_*∈*R^Q^*, with *D* being the dimension of the data space (here: 48), *Q* the dimension of the latent space (usually 2 or 3) and *N* the number of samples in the dataset. Then, probabilistic PCA can be written as
(1)
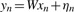

with i.i.d. observation noise *η_n_*: *p*(*η_n_*)=*N*(*η_n_*|0,*β*^−1^*I*) (Bishop, 2006). While for probabilistic PCA, we would marginalize over *X* and optimize the transformation matrix *W,* for GPLVM we marginalize over *W* and optimize the latent variables *X.* If we place a prior over *W* in the form of *p*(*W*) = Π_*i*=1_^*D*^ *N* (*w_i_*|0, *α*^−1^*I*) where *w_i_* is the *i*th row of *W* and integrate over *W* we find (Lawrence, 2004):
(2)
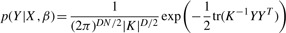

with *K* = *αXX^T^* +*β*^−1^ *I*. This marginalized likelihood is the product of *D* Gaussian processes with linear covariance matrix *K*. If we now substitute the linear kernel in *K* with a different kernel such as an rbf kernel or a rational quadratic kernel, we will yield a GPLVM. We can then learn a latent representation of the data *X* as well as the kernel hyperparameters by optimizing the log-likelihood. The latter can be written as
(3)
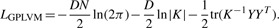

To optimize the log-likelihood, nonlinear optimisers such as scaled conjugate gradient (Nabney, 2001) can be used after having determined the gradient of the log-likelihood with respect to the latent points and the kernel parameters.

To assess the benefit of using a nonlinear dimensionality reduction scheme, we performed GPLVM as well as a PCAon the data. The embeddings were evaluated by calculating the nearest neighbour error in the latent space for the following cell types: 1-cell stage, 2-cell stage,..., 16-cell stage, TE cells, PE cells, ICM cells and EPI cells.

### 2.2 Structure-preserving GPLVM

Although GPLVM facilitates an interpretable nonlinear embedding of the high-dimensional gene-expression data including a gene relevance analysis, it has several drawbacks. Thus, it does not preserve local distances and does not take the structure of the input data into account.

An important characteristic of dimensionality reduction approaches in general, is how the algorithm preserves distances between points in the original data space. Algorithms such as t-SNE (van der Maaten and Hinton, 2008) or Sammon's mapping (Sammon, 1969) find an embedding by preserving local distances (i.e. points which are close together in the data space will be close in the latent space). GPLVM, in contrast, generates a smooth mapping from the latent space to the data space; this implies that two points which are distant in the data space will be distant in the latent space, too. This can be interpreted as preserving dissimilarities (rather than local distances or similarities) (Lawrence, 2006). While it can be desirable to preserve local distances, it is important to not put too much focus on this property as there are two dangers related to the preservation of local distances: first, points that are distant in the data space may be close in the latent space and important differences could be masked. Second, the focus on small local distances leads to a relatively high sensitivity to noise. (Lawrence, 2006) generalize GPLVM to preserve local distances by introducing back-constraints.

Furthermore, an issue exists which is more generally related to using dimensionality reduction methods on data with a known structure, as presented in (Guo *et al.*, 2010). Both linear and nonlinear dimensionality reduction methods such as PCA or GPLVM do not take information on the structure of a dataset into account when generating a mapping. However, when including information on the local structure of the data (i.e. which cells correspond to which cell stage) it is important not to focus too much on this property to avoid an artificial separation of similar data points (e.g., a specific cell type such as TE cells can occur during two different developmental stages which should be reflected in the embedding; i.e,. TE cells from two different cell stages should be allowed to overlap).

In the following, we present a novel approach solving both of the above issues, the lack of preservation of local distances and the consideration of prior knowledge on the structure of a dataset. Therefore, we simultaneously address the common challenge which underlies both issues: in both cases it is desirable to find a trade-off between the benefits of classical GPLVM (preservation of dissimilarities across the whole dataset) and the potential benefits of including local information on local distances as well as local structure. This can be achieved by placing an appropriate prior *p*(*X*) on the embedding *X* which should encourage the preservation of local structure as well as local distance. More formally, we can first define a cost function which minimizes stress within the given sub-structures of the data. This cost function should encourage that data points from the same developmental stage which are close in data space should be close in latent space, too. We chose to use a weighted sum of squares as proposed by (Sammon, 1969), focussing on matching small local distances for data from the same group (i.e., developmental stage).
(4)
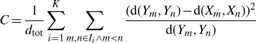

with *K* being the number of substructures and *I_i_* being the set of indices corresponding to all data-points in the *i*th substructure. d(*Y_m_, Y_n_*) are the pairwise distances in the data space and d(*X_m_, X_n_*) the pairwise distances in the latent space;

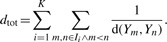

Next, this cost function is used to place a ‘data prior’ *p*(*X*) on *X* :
(5)
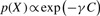

with *γ* being a tuning parameter controlling the influence of the local structure. The effect of different choices of *γ* is discussed in the next sections. Consequently, the log posterior function can be written as
(6)
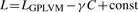

As for the GPLVM, minimization is performed using the Netlab implementation of the scaled conjugate gradient algorithm (Nabney, 2001). This can be interpreted as a regularized GPLVM and is somewhat reminiscent of dynamic GPLVMs (Wang *et al.*, 2006) which allow for embedding motion sequences by placing an appropriate prior on *X* and of discriminative GPLVMs which use a training set to learn a supervised embedding by making use of an LDA-based prior (Urtasun and Darrell, 2007).

Our novel approach satisfies well the requirements needed for embedding high-dimensional gene expression data in a low-dimensional space: first, due to the preservation of dissimilarities, it results in a global representation of the data which facilitates the identification of cell types across different developmental stages. Second, it allows for a reliable identification of subclusters within a developmental stage as the preservation of small local distances ensures that points within a sub-cluster are similar to each other (i.e. the euclidean distance in the data space is small). Third, the nonlinear nature of the embedding will allow for appropriate representation of the potentially complex interrelation between genes while—via the mapping from latent space to data space—ensuring interpretability.

### 2.3 Interpretability of GPLVM-based embeddings

As GPLVMs establish a nonparametric mapping from the latent space to the data space, interpreting mappings is not straight-forward. We introduce relevance maps illustrating which gene is most important at specific locations of the embedding. These maps are generated by first determining for a 20×20 grid in the low-dimensional latent-space the corresponding values in data space. To make a prediction for a new point x* in the latent space, we can make use of the probabilistic mapping from latent-space to data space. As for each point in the latent space, the corresponding point in data space has a Gaussian distribution, it is possible to determine the mean value in the data space ŷ*. From standard Gaussian processes (Rasmussen and Williams, 2006), we find that this can be written as
(7)
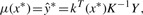

with *k*(x^*^) being the *N* × 1 vector of covariances (*σ*(*x*_1_,x^*^),...*σ*(x_N_,x^*^)) which can be calculated using the previously learnt kernel hyperparameters.

Next, to quantify which variable in the data space (here: which gene) is most important at a specific location in the latent space, we can derive *μ*(*x*) with respect to *x*:
(8)
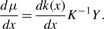

The gradient of the expected value at a number of points in the latent space was calculated. This was done for a 20×20 grid of the entire map and the most important gene (greatest norm of the gradient) was displayed. We term this ‘gene relevance map’. It can be interpreted as a nonlinear generalization of loadings within the linear PCA framework. As for commonly displayed plots of PCA loadings, gene relevence maps can help the user to interpret the embedding by identifying which genes are most important for different regions; for example, this can be particularly helpful for regions corresponding to transitions between different stages/groups as it can help to identify genes which play a key role for this transition.

Furthermore, we calculated the gradient at specific locations in the map to display the change in expression levels of all 48 genes.

### 2.4 Analysed data

Gene expression data from 442 single cells at different developmental stages as presented in Guo *et al.* (2010) were analysed. For each cell stage, the number of analysed embryos varied between 5 and 10; cell numbers (cell stage) were confirmed by counting cells after dissection. As in the original research article, gene expressions were normalized to endogeneous controls by subtracting, for each cell, the average of its Actb and Gapdh levels. The labels for TE, ICM, PE and EPI cells shown in the figures in Section 3 were derived from Figure 1 in (Guo *et al.*, 2010) by assigning each cell to the closest cluster (EPI, TE or PE). Further details on the dataset can be found in Guo *et al.* (2010), specifically in the section ‘Experimental Procedure’.

## 3 RESULTS

### 3.1 Nonlinear method yields a better embedding than linear PCA and ICA

**Fig. 2.**
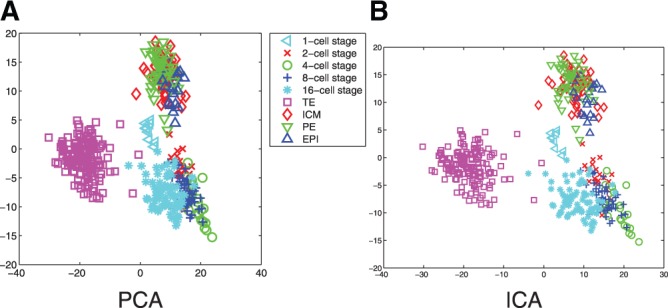
Standard PCA (**a**) and ICA (**b**) for all cells from 1 to 64 cell stage

**Fig. 3.**
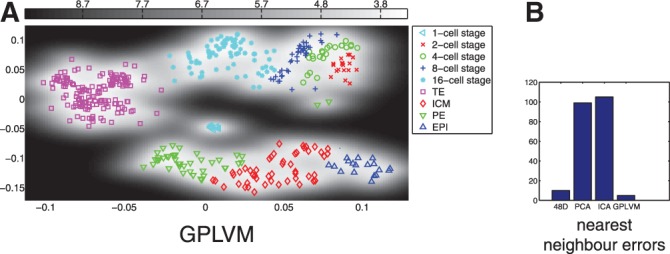
(**a**) GPLVM for all cells from 1 to 64 cell stage. The uncertainty corresponding to the probabilistic mapping from latent space to data space is colour-coded (high SD dark, low SD light); (**b**) nearest neighbour errors for the original high-dimensional space and three embeddings in 2D of all cells from 1 to 64 cell stage

**Fig. 4.**
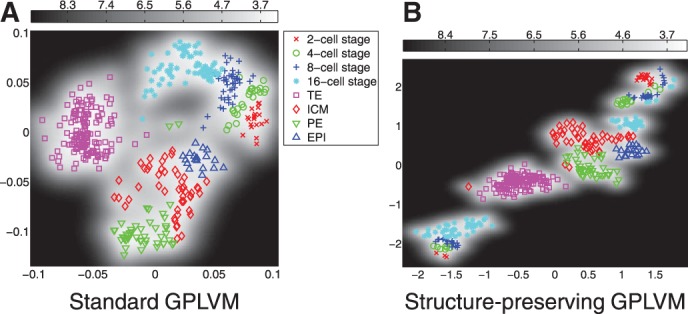
GPLVM for all cells from 2 to 64 cell stage. (**a**) Standard GPLVM. The nearest-neighbour error was 11. (**b**) Structure-preserving GPLVM for all cells from 2 to 64 cell stage with locality parameter *γ*=10^4^ for all cell stages. The nearest-neighbour error was 11

In Figure 2, the results of a PCA and an ICA performed on expressions of all cells from cell stages 1 to 64 are shown. In comparison, Figure 3a shows a nonlinear GPLVM of the same data and illustrates the benefits of including nonlinearities: while in the PCA representation, TE cells and PE cells can be separated, there is a strong overlap for cells from the 1-cell stage to the 8-cell stage. Furthermore, ICM cells and EPI cells are strongly overlapping. In contrast, all cell types and cell stages can be well separated using GPLVM. To quantify the differences in embeddings, we calculated the nearest neighbour error in the latent space for the classes illustrated with different colours in Figures 2 and 3a (1-cell stage, 2-cell stage,..., 16-cell stage, TE cells, PE cells, ICM cells and EPI cells). This resulted in 99 errors for PCA, 105 errors for ICA and 5 errors for GPLVM; when performed in the data space, the nearest neighbour analysis yielded 10 errors (Fig. 3b). It is worth noting the unusual result of a lower number of errors in latent space than in data space. (Guo *et al.*, 2010) point out that due to experimental conditions the data from 1-cell stage are systematically different from the other cells stages; this is reflected in the GPLVM mapping, too. That is why for the subsequent analysis, we only considered data from the 2-cell stage onwards. In Figure 4, the corresponding mapping is shown. It can be seen the mapping separated all cell stages well, with the exception of two outliers. Although being labelled as PE cells, the gene expressions of these cells were significantly different from the gene expressions of all other PE cells. More specifically, when comparing the gene expression levels of the outliers to the other PE cells, Bmp4, Dppa1 and Tspan8 differed strongest, resulting in both cells sharing characteristics with TE cells. Results of univariate analyses of the data and of an hierarchical clustering can be found in Guo *et al.* (2010).

### 3.2 Structure-preserving GPLVM can resolve new sub-populations within developmental stages

**Fig. 5.**
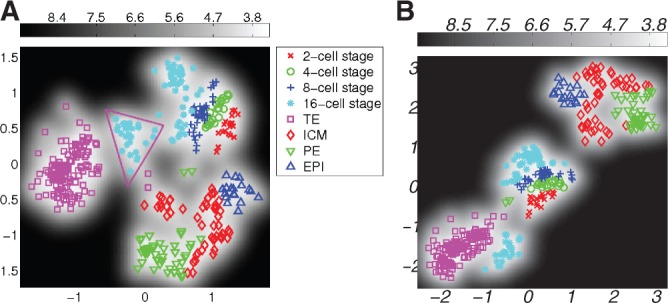
Structure-preserving GPLVM for all cells from 2 to 64 cell stage with different values of *γ.* (**a**) *γ* = 100 for cell stages 2 to 8, *γ* = 15000 for the 16-cell stange and *γ* = 20000 for the 32- and 64-cell stages. Cells assigned to the TE-like subcluster are within the purple triangle. The nearest-neighbour error was 6. (**b**) *γ* = 100 for cell stages 2 to 8, *γ* = 20000 for the 16-cell stange and *γ* = 30000 for the 32- and 64-cell stages. The nearest-neighbour error was 5

**Fig. 6.**
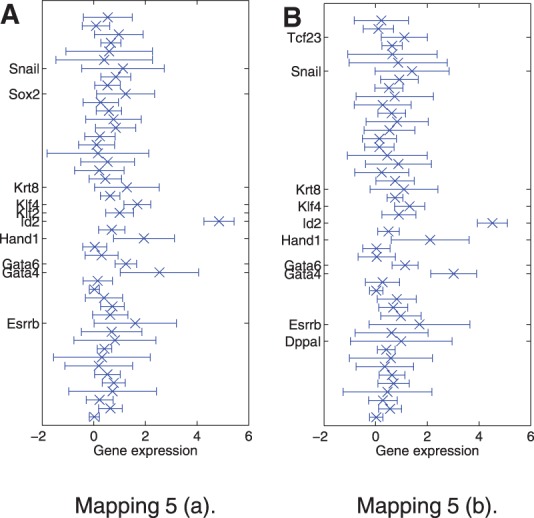
Difference in gene expression between the two subclusters at the 16-cell stage for different mappings. The error bars show the variation of gene expression within the smaller subcluster (1 SD in each direction). For convenience, genes with the strongest differences are labelled in the plots. The order of all genes from top to bottom is Actb, Ahcy, Aqp3, Atp12a, Bmp4, Cdx2, Creb312, Cebpa, Dab2, DppaI, Eomes, Esrrb, Fgf4, Fgfr2, Fn1, Gapdh, Gata3, Gata4, Gata6, Grhl1, Grhl2, Hand1, Hnf4a, Id2, Klf2, Klf4, Klf5, Krt8, Lcp1, Mbnl3, Msc, Msx2, Nanog, Pdgfa, Pdgfra, Pecam1, Pou5f1, Runx1, Sox2, Sall4, Sox17, Snail, Sox13, Tcfap2a, Tcfap2c, Tcf23, Utf1 and Tspan8

**Fig. 7.**
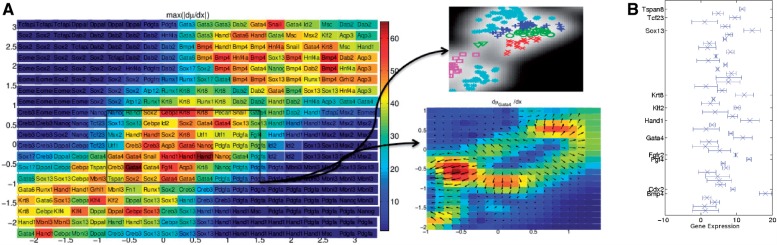
Relevance map showing the greatest norm of the gradient across the entire map (left) and norm of the gradient for all genes at the centre of the ICM cluster (right). (**a**) Gene relevance map corresponding to the mapping in Figure 5b. The region of the map corresponding to early cell stages, including the 16-cell stage is shown in more detail (middle). Here, the gradient of Gata4 with respect to *x* is shown: the colour illustrates the norm of the gradient, the arrows illustrate the direction. It can be seen how between the 8-cell stage and the TE-like subcluster at the 16-cell stage considerably greater changes in Gata4 occur than between the 8-cell stage and the non-TE-like subcluster. For convenince, also the corresponding part of the embedding in Figure 5b is shown (middle, top). (**b**) Gradient at the centre of the ICM cluster; the error bars reflect the uncertainty of the mapping (1 SD in each direction)

To take the structure of the data into account, an additional term is introduced in the likelihood, which encourages the preservation of small distances of data points from the same developmental stage (i.e. 2-cell stage, 4-cell stage,..., 64-cell stage). If the locality parameter is chosen too low, the additional term *C* in the likelihood does not have a visible effect on the embedding; if it is chosen too high, the algorithm focusses too much on small distances and the data are broken up in many isolated small clusters of similar data-points. In Figure 4b, such embedding is illustrated for the locality parameter *γ* = 10^4^. It is also possible to introduce different values of *γ* for different cell stages, reflecting prior knowledge on the variation of the gene expressions within a specific cell stage. As we believe there is little variation within the different stages from two cells to eight cells, we can choose a lower value of *γ* for these data. In contrast, as it is known that at the 32-cell stage and the 64-cell stage, cells have already undergone differentiation into 2 and 3 different cell types, respectively, we can choose a higher value for *γ* for these data to allow for greater heterogeneities. This is illustrated in Figure 5. It can be seen that for all mappings we find a separation of the cells at the 16-cell stage into two sub-clusters; depending on the choice of *γ*, this separation occurs at different degrees in the embedding. In Figure 6, differences in mean gene expression are shown for the two sub-clusters for different mappings. In Figure 6a all cells in the 16-cell stage were assigned to one of two clusters based on a Gaussian mixture model. In Figure 6, it can be seen that the separation was consistent across different embeddings: Id2, Gata4 and, to a smaller extend Hand1 and Klf4, are differentially expressed in the subclusters derived from both mappings shown in Figure 5. Changes in Gata4 between the 8-cell stage and the 16-cell stage are illustrated in detail in Figure 7. This difference in expression levels also corresponded to a smaller distance between the cells in the subcluster closer to the TE region and TE cells than between the cells in the other subcluster and TE cells. This was quantified by calculating the minimum distance between any cell in the 16-cell stage and TE cells. For both mappings, cells located in the subcluster closer to TE cells had a significantly smaller Euclidean distance in data space to TE cells than cells from the other subcluster (*P<*10^−5^ for both mappings, *t*-test). Thus, the separation of cells in the 16-cell stage into two subclusters with one subcluster being significantly more TE-like than the rest of the population is consistent for different choices of the locality parameter.

As the embedding shown in Figure 5b resolves differences in gene expression between the differential stages as well as within the 16-cell stage best, the corresponding mapping was analysed in detail. To assess which genes are most important across the different cell stages, a gene relevance map was generated; the most important genes (i.e., the gene changing most when moving away from a specific position on the map) are shown in Figure 7. In Figure 7b, the gradient at the centre of the ICM cluster is shown. It can be seen that while Bmp4 is the most important gene, Fgfr2/Fgf4, and Hand1 are important as well. This confirms previous findings on the importance of Fgf signalling (Guo *et al.*, 2010) and the relevance of Bmp4 and Gata4 (Coucouvanis and Martin, 1999; Fujikura *et al.*, 2002).

Thus, in spite of the more complex nature of the GPLVM mapping, it is possible to reproduce findings from standard PCA analyses and, in addition to this, identify new subpopulations at early cell stages.

## 4 DISCUSSION

Using a nonlinear embedding allows to assess differences in gene expression patterns, both within the same developmental stages and across different developmental stages which cannot be resolved with linear PCA. We have presented a framework which combines the advantages of nonlinear dimensionality reduction methods with interpretability and the ability to take prior knowledge on the structure of the data into account. This was achieved by taking advantage of the smooth mapping within GPLVMs as this allows for the construction of relevance maps which illustrate which genes are most important at different stages of the embryonic development. Furthermore, an additional term in the likelihood of GPLVM was introduced which encourages the preservation of small local distances between data points of the same developmental stage. This facilitates a better discovery of novel subclasses within the same developmental stage. Although we have presented this framework at the example of single-cell gene expression data of the developing mouse embryo, our algorithm is suitable for a wider range of problems: our result indicates that it may be beneficial for datasets consisting of single-cell gene expression data from different groups of cells, when at least one of the groups is expected to be heterogeneous. For example, this can be the case for other types of stem cells such as blood stem cells from different developmental stages or tumour cells from different clinical or pathological stages.

The separation of the 16-cell stage in two distinct subclusters could be discovered using the extended GPLVM; it could be shown that the cells assigned to one of the subclusters were significantly more similar to TE cells than the cells in the other subcluster. This indicates that these cells may be more likely to become TE cells than ICM cells in subsequent stages of the cellular development. When analyzing differences in the gene expressions patterns of the two newly identified subpopulations, the strongest difference occurred for Id2. This is consistent with the findings in (Guo *et al.*, 2010) who report that Id2 is the earliest TE-specific marker; however, we also found considerable differences in the levels of Gata4 and, to a smaller extent, Klf4 and Hand1. Although (Guo *et al.*, 2010) identified subpopulations with high/low Id2 at the 16-cell stage via a univariate violin-plot, we have shown that these sub-populations can also be resolved using the extended GPLVM framework. This has the advantage that the differences in gene expressions across all 48 genes can be identified simultaneously, rather than changes in one gene only. Thus, it can be seen from the analysis of the sub-clusters that not only Id2 is differentially expressed but also Gata4; this suggests that not only Id2 but also the expression level of Gata4 at the 16-cell stage could indicate whether a cell is more likely to become a TE cell or an ICM cell. Similarly, Hand1, which is a known marker for TE cells (Cross *et al.*, 1995; Strumpf *et al.*, 2005), was differentially expressed between the two subclusters in the 16-cell state. Furthermore, a GPLVM-based approach to identify new subpopulations scales well to higher dimensions. Although it is possible to analyse univariate violin-plots for 48 genes, this can become impractical when a very high number of genes is analysed.

Although the application of the extended GPLVM algorithm allows for a clear separation of two subclusters in the 16-cell stage, the parameter *γ* has to be chosen carefully; to find an adequate value resulting in a mapping with the right balance of preserving dissimilarities as well as local distances within developmental stages, it can be necessary to vary *γ* over a wide range which may be time-consuming. In general, when trying to find an appropriate value for *γ,* it can be helpful to start off with standard GPLVM (*γ* = 0). Next, *γ* can be increased iteratively until the data are broken up in many small, isolated clusters of similar data-points; in this case (as illustrated in Fig. 5 for early cell stages), too much weight is placed on preserving small local distances.

Depending on the application of the algorithm, it may also be beneficial to use a different distance measure or a different cost function to encourage the preservation of local distances. Although we have used Euclidean distances to calculate the distances in the data space, it is also possible to use measures such as a correlation coefficient or the L1 measure. Furthermore, different cost functions instead of Sammon's stress could be used. For example, it is possible to use the t-SNE cost function, which first converts distances in data space and latent space into probabilities and then minimizes the KL divergence for probabilities in data and latent space (van der Maaten and Hinton, 2008). For our application, we have chosen Sammon's stress as it focuses mostly on preserving small distances; large dissimilarities are preserved within the GPLVM framework which allows for an intuitive control of the trade-off between the local preservation of distances within a cell stage and the global preservation of dissimilarities via *γ*.

Recently, a variety of other nonlinear embeddings have been proposed, such as locally linear embedding (LLE) (Roweis and Saul, 2000), isomap (Tenenbaum *et al.*, 2000), t-SNE (van der Maaten and Hinton, 2008), tree-preserving embedding (TPE) (Shieh *et al.*, 2011) or autoencoders (Hinton and Salakhutdinov, 2006). While in principle any of these algorithms could be used to find a low-dimensional embedding for high-dimensional gene-expression data, they all have drawbacks. Thus, like Sammon's mapping, LLE and isomap focus on the preservation of local distances; as discussed in Section 2.2, this has the drawback that important dissimilarities may be masked and the disadvantage a high sensitivity to noise. These drawbacks are addressed in the t-SNE and TPE algorithms, but both techniques do not result in explicit mappings between latent space and data space and thus lack interpretability. Although such explicit mappings can be generated with autoencoders, they share an important drawback with the other techniques as it is not clear how the group structure of the data can be taken into account. That is why we chose to use GPLVM-based embeddings.

A major weakness of the GPLVM-based approach is that it is computationally expensive. Although computation times were still feasible for the data presented here (~1 h on a standard PC), this method may be prohibitive for very large data sets. However, in this case, sparse approximations as for Gaussian process regression can be applied. Applying these reduced rank approximations to the covariance matrix results in significant speedups so that also large data sets can be analysed on reasonable time scales as the computational complexity decreases from *O*(*N*^3^) to *O*(*k*^2^*N*), with *N* being the number of points in the dataset and *k* being the number of so-called active points (typically about 100) (Lawrence, 2007). Thus, the GPLVM framework is a valuable tool for analyzing RNA sequencing data from single-cell studies—which are a promising and increasingly popular experimental technique—as typical challenges for this type of data include the analysis of many features and generating a mapping with uncertainty. However, other interpretability challenges which arise from working with gene expression data (as here) and smaller sequence parts (when moving towards NGS techniques) remain to be addressed.

## 5 CONCLUSION

We have presented a novel framework for resolving differences in gene expression patterns for the early mouse embryo based on single-cell gene expression data. Therefore, a nonlinear mapping between a low-dimensional latent space and the high-dimensional data space was combined with gene relevance maps and gradient plots in order to ensure interpretability. A novel extension to the GPLVM algorithm taking the local structure of the data into account and preserving small local distances within the same developmental stage was presented. Using this new approach, we could resolve differences of gene expressions between all cell stages as well as identify a new sub-population at the 16-cell stage with was significantly more TE-like than other cells at the 16-cell stage.

*Funding:* CoReNe and the ERC.

*Conflict of Interest:* none declared.
